# Enhancing the biodiesel production in the green alga *Chlorella vulgaris* by heavy metal stress and prediction of fuel properties from fatty acid profiles

**DOI:** 10.1007/s11356-024-33538-w

**Published:** 2024-05-14

**Authors:** Hani Saber, Hamdy Ramadan Galal, Mohamed Abo-Eldahab, Eman Alwaleed

**Affiliations:** Department of Botany and Microbiology, Faculty of Science, South Valley University, Qena, 83523 Egypt

**Keywords:** Bioenergy, Microalgae, Fatty acid, Lipid, Relative increase in energy compound, Heavy metals stress

## Abstract

The green microalga *Chlorella vulgaris* was used as a test organism during this study for evaluation of the impact of different heavy metal stress, Mn^2+^, Co^2+^, and Zn^2+^, on enhancing the biodiesel production. The algal cultures were grown for 13 days under heavy metal stress after which were subjected to estimation of growth, some primary metabolites, lipid, and fatty acid profiles. The maximum lipid accumulation (283.30 mg/g CDW) was recorded in the algal culture treated with 3 µM cobalt nitrate. Application of 2 mM manganese chloride; 1, 2, and 3 μM cobalt nitrate; and 0.2, 0.4, and 0.6 mM zinc sulfate caused highly significant increases in the lipid contents amounting to 183.8, 191.4, 230.6, 283.3, 176.3, 226.0, and 212.1 mg/g CDW, respectively, in comparison to control (153.4 mg/g CDW). The maximum proportion of saturated fatty acids (SFA) (64.44%) was noted in the culture treated with 6 mM MnCl_2_ due to the existence of palmitic acid (C16:0), stearic acid (C18:0), and pentadecylic acid (C15:0) which are represented by 53.59%, 5.96%, and 1.37%, respectively, of the total FAs. Relative increase in energy compound (REEC) showed that 1, 2, and 3 µM Co^2+^ lead to the highest stimulation in lipid and carbohydrate contents to 0.207, 0.352, and 0.329 × 10^3^%, respectively. Empirical formulas were used for the assessment of biodiesel fuel properties based on FAME composition. The estimated properties met the prescribed international standard criteria.

## Introduction

Fossil fuels that emit carbon dioxide and other greenhouse gases are dominated by new and emerging energy systems (Bórawski et al. 2019). The planet needs a major and coordinated transformation of its energy supplies to reach our global climate goals and prevent harmful climate change. So, we need to substitute fossil fuels with an alternate renewable, affordable, and environment-friendly energy source (Ansari et al. 2020). Biodiesel is a prospective fuel for substitution of the traditional fossil fuels. Due to the food versus fuel dilemma, the first and second generations of biofuel could not develop themselves as viable biodiesel source output. One of the promising, satisfying, and ever-increasing demands for transport fuel has been identified as biodiesel from microalgae (Chisti 2007). Nineteen to 57.000 L oil/acre can be produced by microalgae in 1 year better than any other preceding biodiesel source (Demirbas and Demirbas 2011). Other valuable constituents found in the residual biomass of algae like polysaccharides, proteins, and remaining lipids could be useful in the production of bio-oil, bio-ethanol, bio-hydrogen, and bio-gas (Dębowski et al. 2013).

Since they operate as cofactors in metal enzymes and as precursors to vitamins, heavy metals are vital micronutrients for all biota (Munda and Hudnik 1988). Despite this, high levels of heavy metals can reduce the production of chlorophyll, alter photosynthetic activity, and cause lipid peroxidation in algae by forming reactive oxygen species (ROS) in the cells of algae (Siripornadulsil et al. 2002). The amount of oxidized protein and lipid in the algal cell is suggestive that many microalgal organisms suffer from stress (Tripathi and Gaur 2006). Furthermore, polyunsaturated fatty acids (PUFA) can be overproduced by cells that are resistant to growth inhibitors (Fatma and Sultan 1999). Zinc is an essential micro-nutrient well known for natural algae growth. Its shortage results in a slower growth rate (Shrotri et al. 1981). Whitton (1970) elucidated the tolerance and growth response of different algae to Zn^2+^ and explained that lower metal concentrations will encourage algal growth while higher levels will entirely prohibit it. Most creatures require manganese, which is an essential micronutrient. It contributes to the design of proteins and enzymes involved in photosynthetic processes in algae. The excess tends to destroy the photosynthetic apparatus in particular (Mukhopadhyay and Sharma 1991). Manganese serves two purposes in the metabolic process as an important micronutrient at low levels and as a harmful component at greater concentrations (Dučić and Polle 2007). Low cobalt concentrations increased the efficacy of the antioxidants, while higher levels had detrimental effects (Myśliwa Kurdziel et al. 2004). So, this work was intended for screening and evaluating the lipid productivity and fatty acid profile of *Chlorella vulgaris* and enhancing the biodiesel production of the investigated alga by different heavy metal stress. In addition, predicting the biodiesel properties from the fatty acid profile and calculating the relative increase in energy compound (REEC %).

## Materials and methods

### Experimental layout

*Chlorella vulgaris*, a freshwater green microalga, was isolated from the water of the River Nile at Qena, Egypt, and chosen to evaluate its lipid content as a potential source for biodiesel production and produce high-quality biofuel under heavy metal stress conditions. Algal cells were isolated and repeatedly subcultured on a solid bold basal medium (BBM) (Nichols 1973) before being transferred to a sterilized liquid nutrient medium. Eighty milliliters of the *C. vulgaris* culture was inoculated into an 8-L, sterilized clear polyethylene tank of BBM, and the tank was then incubated at 25 ± 2 °C. With a 16:8 light:dark photoperiod and a light intensity between 92 and 95 µ mol m^−2^ s^−1^, cool white fluorescent lighting (Philips Master TL-D 85 W/840) was used to illuminate the cultures for 13 days. After preliminary experiments on treating the algal cultures with heavy metals, *C. vulgaris* was exposed to various concentrations of manganese chloride (2, 4, and 6 mM), cobalt nitrate (1, 2, and 3 μM), and zinc sulfate (0.2, 0.4, 0.6, and 0.8 mM) after which, growth, some primary metabolites, lipid contents, and fatty acid profile were assessed.

#### Estimation of growth (optical density, biomass, and dry weight)

Spectrophotometric optical density measurements at 450 nm (OD_450_) were used to measure the growth of *C. vulgaris* (Battah et al. 2015; El Agawany and Kaamoush 2022). According to Abomohra et al. (2013), biomass production was evaluated by determining the algal cellular dry weight (CDW, g L^−1^) using the following formula (Eq. 1):

1 $$\mathrm{Biomass\;productivity\;}(\mathrm{CDW\;g\;L}\;-\;1\;{{\text{day}}}^{-1})\;=\;({{\text{CDW}}}_{{\text{L}}}-\;{{\text{CDW}}}_{0})/({{\text{T}}}_{{\text{L}}}\;-\;{{\text{T}}}_{0})$$

where CDW_**0**_ refers to the CDW at the early exponential phase (*T*_0_) and CDW_L_ at the late exponential phase (*T*_L_).

For the estimation of algal dry weight, a weighted membrane filter was used to filter an aliquot of the algal suspension. After precipitating on the filter, the cells were twice rinsed with distilled water and then left to dry overnight at 105 °C. The algal dry weight was estimated as mg mL^−1^ of algal suspension (Leganés et al. 1987).

#### Estimation of pigments

According to Marker (1972), pigment fractions were estimated spectrophotometrically (T60 UV- visible spectrophotometer). The formulas developed by Metzner et al. (1965) were used to determine the pigment fraction content (µg/mL algal suspension).

#### Estimation of carbohydrate and protein

The anthrone-sulfuric acid method (Yemm and Willis 1954) was used to determine the carbohydrate contents. The amount of protein was determined according to Lowery et al. (1951).

#### Lipid extraction

With some modifications, the method of Folch et al. (1957) was used to extract total lipids. A definite dried algal biomass (1 g) was homogenized with a solvent mixture ratio of chloroform:methanol 1:2, in a glass homogenizer, and washed with a 0.9% (w/v) NaCl solution water for 2 min and stirred for 4 h at room temperature. After that 1 mL of chloroform was added and mixed for 30 s. One more milliliter of distilled water was added, and mixing was carried out for an additional 30 s. Then the suspension was centrifuged (32 × 10^3^ RCF, 4 °C, 10 min) and allowed for separation into three layers. The methanol/NaCl 0.9% were discarded. The chloroform layer was collected. The residual biomass was extracted twice more. The chloroform extracts were pooled and kept at 4 °C overnight. The collected chloroform extracts’ volume was measured and the non-lipid components (pigments and lipoproteins) were removed (Vogel 1975). The lipid extracts were placed in a pre-weighted glass vial, which was then dried under an argon stream, incubated at 80 °C for 30 min, cooled in a desiccator, and the pooled chloroform extracts were weighted, the total lipid contents were calculated using the following formula (Eq. 2) (John et al. 2011):

2 $$\mathrm{Total\;lipid }\;(\mathrm{\%})\;=\;\mathrm{ Lipid\;weight}/\mathrm{algae\;total\;weight\;}\times\;100$$

#### Transesterification

The isolated lipids were saponified with ethanolic KOH (20%, w/v) at room temperature for the entire overnight period. By acidifying with 5 N hydrochloric acid and extracting with petroleum ether at 40–60 °C, fatty acids were freed from their potassium salts. Fatty acid methyl esters were present in the ether extract, which was cleaned three times with distilled water before being dried for a whole night with anhydrous sodium sulfate (Furniss et al. 1989).

#### Characterization of trans-esterified algal oil using GC-MS

The fatty acid profile was analyzed using a Trace GC1310-ISQ mass spectrometer (Thermo Scientific, Austin, TX, USA). By comparing the components’ retention times and mass spectra to those in the WILEY 09 and NIST 11 mass spectral databases, the components were identified (Pandey et al. 2010).

The following formula (Eq. 3) was used to compute the percentage of each fatty acid (Abomohra et al. 2013),

3 $$\mathrm{Fatty\;acid\;proportion\;}= F/{F}_{{\text{t}}}\times\;100$$

where *F* is the desired fatty acid’s peak area and *F*_t_ is the total fatty acid methyl ester peak area.

#### Prediction of fuel properties from fatty acid profiles

Several equations have been suggested to predict the fuel properties based on fatty acid composition (Francisco et al. 2010; Nascimento et al. 2013) such as cetane number (CN) (Eq. 4), saponification value (SV) (Eq. 5), iodine value (IV) (Eq. 6), degree of unsaturation (DU) (Eq. 7), long-chain saturation factor (LCSF) (Eq. 8) and cold filter plugging point (CFPP) (Eq. 9) (Nascimento et al. 2013), oxidative stability (OS) (Eq. 10) (Knothe 2007), cold-flow properties like cloud point (CP) (Eq. 11), pour point (PP) (Eq. 12) (Sarin et al. 2009), and flash point (FP) (Eq. 13) (Agarwal et al. 2010). International biodiesel standards such as European (EN 14214), American (ASTM D6751), and Indian (IC15607) provide biodiesel fuel specifications (Hoekman et al. 2012).

4 $$\mathrm{CN\;}= 46.3\;+\;(\frac{5448}{SV})-(0.005\times\;IV)$$

5 $$\mathrm{SV\;}=\;\sum\;(\frac{560\;\times\;N}{M})$$

6 $$\mathrm{IV\;}=\;\sum\;(\frac{254\;\times\;DN}{M})$$

where *D* indicates the number of double bonds, *M* denotes FA molecular mass, and *N* represents the percentage of each FA component.

7 $${\text{DU}}=\sum\;MUFA+\left(2\;\times\;PUFA\right)$$

8 $$\mathrm{LCSF\;}\left(0.1\;\times\;{\text{C}}16\right)\left(0.5\;\times\;{\text{C}}18\right)\left(1\;\times\;{\text{C}}20\right)\left(1.5\;\times\;{\text{C}}22\right)+(2\times\;C24)$$

9 $$\mathrm{CFPP\;}= (3.1417\;\times\;LCSF)\;-\;16.477$$

10 $$\mathrm{OS }\;=\;-\;0.0384\;\times\;\mathrm{\;DU\;}\;+\;7.770$$

11 $$\mathrm{CP\;}=\;(0.526 \times\;\mathrm{\;C}16)\;-\;4.992$$

12 $$\mathrm{PP }= \left(0.571 \times \mathrm{ C}16\right)-12.240$$

13 $$\mathrm{FP\;}\;=\;205.226\;+\;\left(0.083\;\times\;{\text{C}}16:\;0\right)\;-\; \left(1.723\;\times\;\mathrm{\;C}18:0\right)\;-\;\left(0.5717\;\times\;{\text{C}}18:\;1\right)\;-\;\left(0.3557\;\times\;{\text{C}}18:\;2\right)\;-\; \left(0.46\mathrm{\;C}18: 3\right)\;-\;(0.2287\;\times\;{\text{C}}22)$$

#### Selection of the promising stimulated heavy metals

Selection of the promising heavy metals led to stimulation in the energy compound of *C. vulgaris* depends primarily on lipid and carbohydrate production, both of which are regulated by biomass yield. However, it is possible that the heavy metals that cause high lipid production are not the same ones that cause high carbohydrate productivity. As a result, a novel-developed equation (Eq. 14) was used for selection based on the relative increase in energy compounds (REEC, %) (Osman et al. 2020) as follows:

14 $$\mathrm{REEC }\;(\mathrm{\%})\;=\;[{ \left(\frac{{P}_{H}\;-\;{ P}_{L}}{{P}_{L}}\right)}_{carb.}+{\left(\frac{{P}_{H}\;-\;{ P}_{L}}{{P}_{L}}\right)}_{lip.}]\times 100$$

where *P*_H_ refers to the production of carbohydrates (Carb) and lipids (Lip) for the investigated species, while *P*_L_ indicates the lowest recorded productivities among the investigated heavy metals.

### Statistical analysis

Results are shown as the mean and standard deviation (SD) of three replicates. Using the SPSS program (version IBM 25), the collected data were statistically analyzed to assess their degree of significance using one-way analysis of variance (ANOVA), followed by Duncan post hoc testing at probability level (*P*) ≤ 0.05.

## Results

**Fig. 1:**
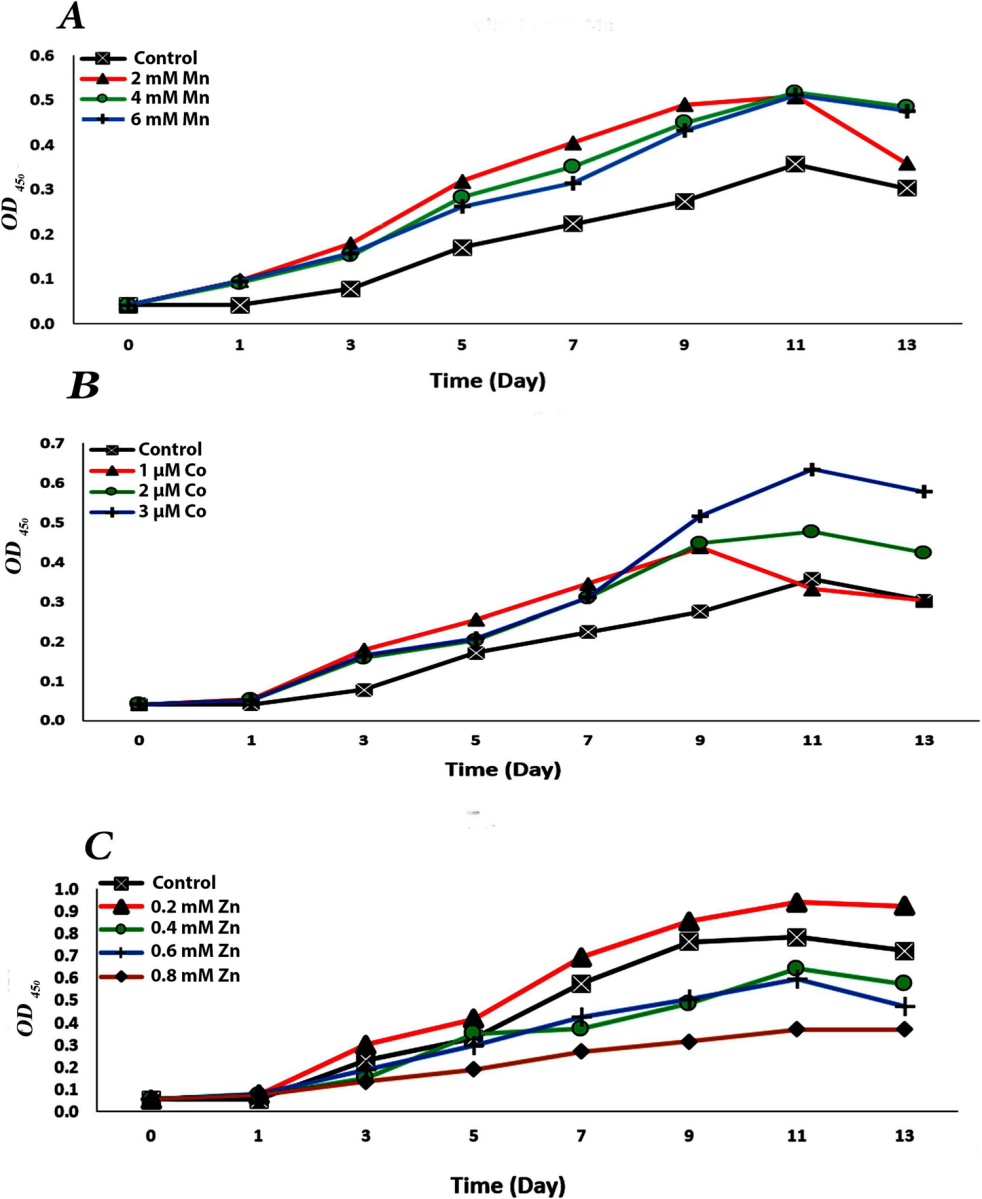
Effect of different concentrations of manganese chloride (**A**), cobalt nitrate (**B**), and zinc sulphate (**C**) on growth measured as optical density of *Chlorella vulgaris*

**Fig. 2:**
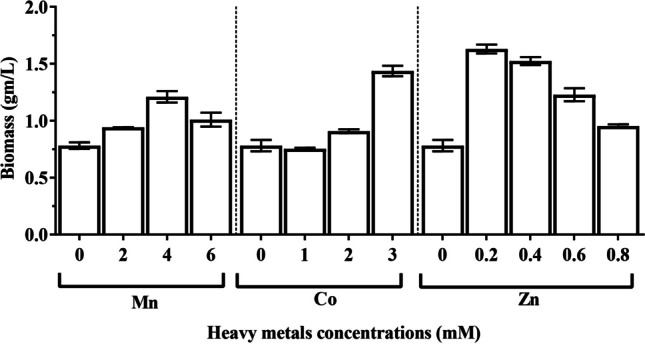
Effect of different concentrations of manganese chloride, cobalt nitrate, and zinc sulfate on biomass productivity of *Chlorella vulgaris*

**Fig. 3:**
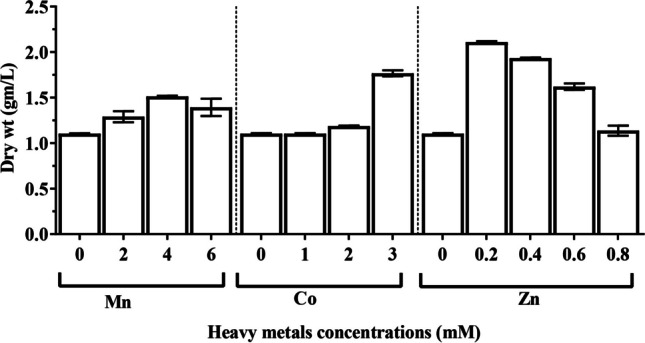
Effect of different concentrations of manganese chloride, cobalt nitrate, and zinc sulfate on growth measured as dry weight of *Chlorella vulgaris*

*Chlorella vulgaris* cultures were exposed to various concentrations of heavy metal, including MnCl_2_ (2, 4, and 6 mM), Co(NO_3_)_2_∙6H_2_O (1, 2, and 3 µM), and ZnSO_4_∙7H_2_O (0.2, 0.4, 0.6, and 0.8 mM) to assess their impacts on growth and metabolic activities. The stationary phase of *C. vulgaris* varied between 11 and 13 days. The growth curve (Fig. 1) demonstrates that the optical density reached its maximum value (0.940) after 11 days of cultivation in the algal culture exposed to 0.2 mM ZnSO_4_∙7H_2_O compared to the untreated control (0.784), while the optical density reached its lowest value (0.302) after 13 days of treatment with 1 µM Co (NO_3_)_2_∙6H_2_O. On the other hand, the treatment of culture with 0.2 mM ZnSO_4_.7H_2_O produced the highest value of *C. vulgaris* biomass production (1.63 CDW g. L^−1^). However, after 11 days of cultivation, the culture treated with 1 µM Co(NO_3_)_2_∙6H_2_O had a low biomass production value (0.75 CDW g. L^−1^) (Fig. 2). In addition, Fig. 3 illustrates how *C. vulgaris* dry weight changed after being exposed to various heavy metal concentrations throughout the stages of the exponential phase. The findings show that after 11 days of incubation, the control culture’s dry weight reached 1.10 mg/mL. Treatment with 0.2 mM ZnSO_4_∙7H_2_O showed the maximum enhancing effect of heavy metal on the dry weight, increasing to 2.11 mg/mL, while the minimum value of dry weight (0.97 mg/mL) was observed at 1 µM Co(NO_3_)_2_.6H_2_O.

### Photosynthetic pigments

**Fig. 4:**
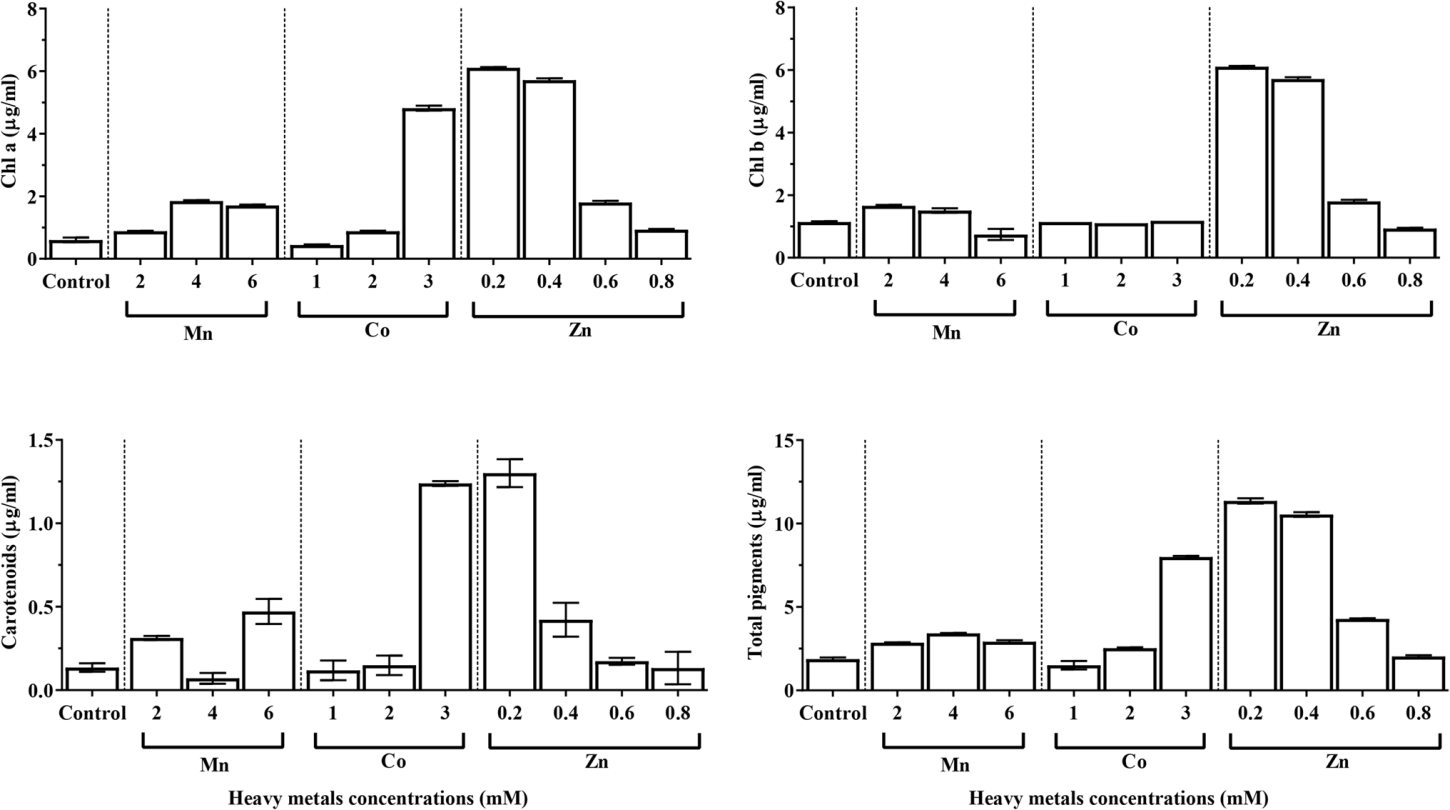
Effect of different concentrations of manganese chloride, cobalt nitrate, and zinc sulfate on photosynthetic pigment of *Chlorella vulgaris*

Increased concentrations of heavy metals had a significant impact on the pigments of *C. vulgaris* (Fig. 4). The chlorophyll a content of *C. vulgaris* increased from 0.601 μg/mL in the untreated culture to 6.11 μg/mL after treatment with 0.2 mM ZnSO_4_∙7H_2_O. Chlorophyll b concentration was 1.14 μg/mL in the untreated culture and rose to 5.22 μg/mL with the addition of 0.4 mM ZnSO_4_∙7H_2_O. The concentration of carotenoids ranged from 0.14 μg/mL in the untreated culture to 1.3 μg/mL at a concentration of 0.2 mM ZnSO_4_∙7H_2_O. Changes in the dosage of heavy metals generally had considerable influences on the total pigment of *C. vulgaris*.

### Carbohydrate and protein contents

**Fig. 5:**
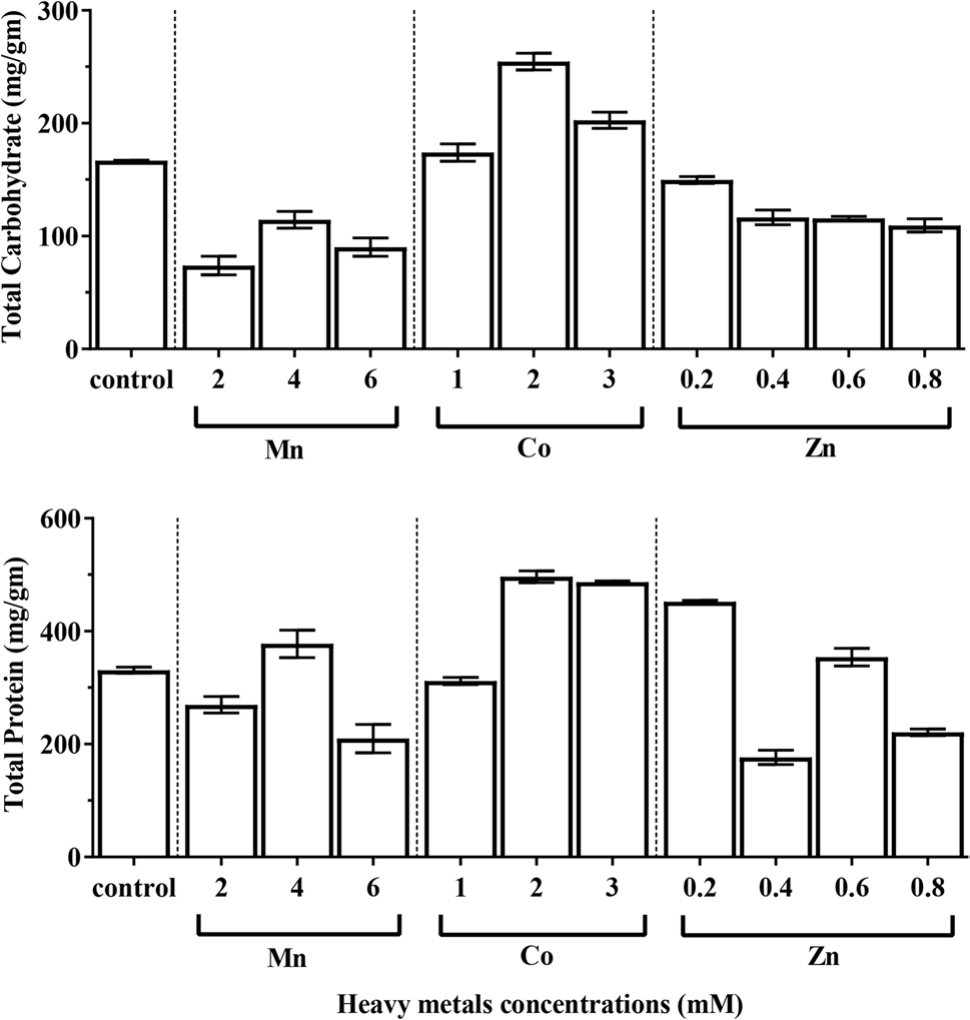
Effect of different concentrations of manganese chloride, cobalt nitrate, and zinc sulfate on carbohydrate and protein contents of *Chlorella vulgaris*

Carbohydrates are the most crucial component of metabolism. Alterations in carbohydrates of *C. vulgaris* under the influence of various heavy metal concentrations during the experiment period were followed (Fig. 5). The highest content of total carbohydrate (254.69 mg/g CDW) was noticed at the end of the incubation period in the algal culture after treatment with 2 µM Co (NO_3_)_2_∙6H_2_O which was 52.55% higher than control. The lowest value of total carbohydrate content (73.91 mg/g CDW) was recorded by application of 2 mM MnCl_2_ which was 56.16% lower than the control value. The response of this green alga in terms of protein content to different heavy metal concentrations is shown in Fig. 5. The total protein revealed a highly significant increase in culture treated with 2 µM Co (NO_3_)_2_∙6H_2_O reaches 496.56 mg/g CDW (50.02% increase over the control value), while the lowest value of total protein was 176.60 mg/g CDW and recorded by application of 0.4 mM ZnSO_4_∙7H_2_O to the algal culture.

### Lipid contents

**Fig. 6:**
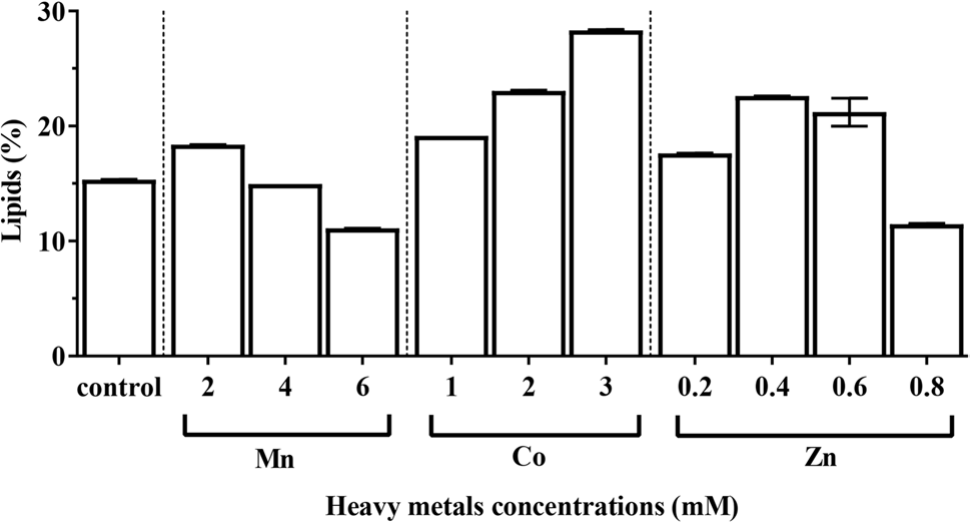
Effect of different concentrations of manganese chloride, cobalt nitrate, and zinc sulfate on lipid content of *Chlorella vulgaris*

From the results in Fig. 6, it could be concluded that the application of different heavy metal concentrations led to a remarkable change in the lipid production of *C. vulgaris*. The maximum lipid accumulation (283.30 mg/g CDW) was recorded in the algal culture treated with 3 µM cobalt nitrate at the end of the experiment. Application of 2 mM MnCl_2_ and 1, 2, and 3 μM Co(NO_3_)_2_∙6H_2_O and 0.2, 0.4, and 0.6 mM ZnSO_4_∙7H_2_O caused highly significant increases in the lipid contents amounting to 183.8, 191.4, 230.6, 283.3, 176.3, 226.0, and 212.1 mg/g CDW, respectively, in comparison to control (153.4 mg/g CDW). On the other hand, a significant decrease in total lipid to 149.6, 111.0, and 114.6 mg/g CDW was recorded in cultures treated with 4, 6 mM MnCl_2,_ and 0.8 mM ZnSO_4_∙7H_2_O, respectively, as compared with the control at the end of cultivation period. Merging carbohydrates and lipid productivities into consideration, the results of relative increase in energy compounds (REEC, %) showed that 1, 2, and 3 µM Co^2+^ led to the highest stimulation in lipid and carbohydrate content in *C. vulgaris*-treated culture (0.207, 0.352, and 0.329 × 10^3^ REEC %, respectively), where carbohydrates reach to 174.05, 254.69, and 202.61 mg/g dry wt., respectively as compared with control (166.96 mg/g dry wt.), and lipid increased to 19.14, 23.06, and 28.33% DW, respectively, as compared to control (15.34 % DW).

### Gas chromatography/mass spectrometry (GC/MS)

Analysis of the chemical composition of algal extracts using GC-MS released numerous compounds which identified after the esterification of lipid and fatty acids. The fatty acid composition in *C. vulgaris* treated with various concentrations of heavy metals was evaluated during the stationary growth phase (Table 1). In general, 20 saturated fatty acids (SFA), 4 monounsaturated fatty acids (MUFA), and 4 polyunsaturated fatty acids (PUFA) were identified. The dominant fatty acids were palmitic acid (16-carbon fatty acid) and stearic acid (18-carbon fatty acid), which revealed noticeable differences in their content with various heavy metal concentrations. Treatment with Zn, Co, and Mn resulted in a remarkable increase in saturated fatty acid contents (SFA). Both total saturated fatty acids (SFA) and polyunsaturated fatty acids (PUFA) are enhanced under heavy metals stress. In addition, total monounsaturated fatty acids (MUFA) increased in the algal culture exposed to different heavy metal concentrations except for 3 µM Co, and 4 and 6 mM Mn, which show decreases in comparison to control. The results showed that the control culture of *C. vulgaris* is characterized by a high percentage of total SFAs reaching 44.56% in comparison to MUFA (28.01%) and PUFA (ND) due to the presence of C16:0 and C18:0, which is represented by 33.72 and 10.84%, respectively, of the total fatty acid. The highest percentage of SFA (64.44%) was recorded in the culture treated with 6 mM MnCl_2_ due to the occurrence of palmitic acid (C16:0), stearic acid (C18:0), and pentadecylic acid (C15:0) which represented by 53.59%, 5.96%, and 1.37%, respectively, of the total FAs. In addition, the application of different heavy metal concentrations induced a highly significant increase in the SFA to 54.60, 51.20, 58.17, 50.34, 54.35, 47.61, 63.13, 48.76, 61.38, and 64.44% of the total fatty acids at 0.2, 0.4, 0.6, and 0.8 mM ZnSO_4_∙7H_2_O; 1, 2, and 3 µM of Co (NO_3_)_2_∙6H_2_O; and 2, 4, and 6 mM of MnCl_2_, respectively. The largest amount of MUFA was 46.56% and noted in the algal culture treated with 2 mM MnCl_2_ due to the presence of oleic acid (C18:1 n-9) and palmitoleic acid (C16:1 n-7) which was represented by 42.17% and 3.57%, respectively. Furthermore, the highest total PUFA was 17.25% of the TFA and recorded at 0.2 mM ZnSO_4_∙7H_2_O due to the presence of stearidonic acid C18:4 n-3 (10.15%) and linoleic acid C18:2 n-6 (6.78%). Finally, from the data, it can be observed that palmitic acid (C16:0) and stearic acid (C18:0) are the most abundant SFA, and oleic acid (C18:1 n-9) and palmitoleic acid (C16:1 n-7) are the most abundant MUFA, while stearidonic acid (C18:4 n-3), linoleic acid (C18:2 n-6), and arachidonic acid (C20:4 n-6) are the most abundant PUFA. Also, C14:0, C20:1, and C18:4 n-3 fatty acids were not detected (ND) in the untreated culture but significantly enhanced under stressed culture conditions. The increased heavy metal concentration resulted in higher production of SFA. Besides, the accumulation of MUFA was enhanced under most heavy metal concentrations. In conclusion, the application of heavy metals on the fatty acid profile of *C. vulgaris* increased the SFA and MUFA in the treated culture in comparison to the untreated control culture which leads to the enhancement of the quantity and the quality of biodiesel.

**Table 1:** Fatty acid profiles of *Chlorella vulgaris* treated with the different concentrations of heavy metals

Common name	FAME	Control	Zn (mM)				Co (µm)			Mn (mM)		
			0.2	0.4	0.6	0.8	1	2	3	2	4	6
Butyric acid Valeric acid Caproic acid Caprylic acid Pelargonic acid Capric acid Undecylic acid Lauric acid Tridecylic acid Myristic acid Pentadecylic acid Palmitic acid Margaric acid Stearic acid Nonadecylic acid Arachidic acid Behenic acid Lignoceric acid Montanic acid Melissic acid Psyllic acid	**C4:0** **C5:0** **C6:0** **C8:0** **C9:0** **C10:0** **C11:0** **C12:0** **C13:0** **C14:0** **C15:0** **C16:0** **C17:0** **C18:0** **C19:0** **C20:0** **C22:0** **C24:0** **C28:0** **C30:0** **C32:0**	**ND** **ND** **ND** **ND** **ND** **ND** **ND** **ND** **ND** **ND** **ND** **33.72** **ND** **10.84** **ND** **ND** **ND** **ND** **ND** **ND** **ND**	**ND** **ND** **0.35** **0.21** **0.22** **0.70** **ND** **1.21** **1.37** **5.14** **3.20** **33.92** **2.69** **3.68** **0.86** **0.10** **0.60** **0.24** **ND** **0.11** **ND**	**ND** **ND** **ND** **ND** **ND** **ND** **0.25** **ND** **ND** **6.75** **1.12** **35.00** **ND** **7.15** **ND** **0.93** **ND** **ND** **ND** **ND** **ND**	**ND** **ND** **ND** **ND** **ND** **ND** **ND** **ND** **ND** **ND** **ND** **58.17** **ND** **ND** **ND** **ND** **ND** **ND** **ND** **ND** **ND**	**ND** **ND** **ND** **0.10** **0.08** **ND** **ND** **ND** **ND** **1.20** **ND** **41.58** **ND** **7.35** **ND** **ND** **0.05** **ND** **ND** **ND** **ND**	**ND** **0.03** **0.22** **0.03** **0.23** **0.23** **0.23** **0.50** **0.0.8** **4.12** **ND** **39.15** **1.37** **7.15** **ND** **0.22** **0.37** **0.03** **0.10** **0.06** **ND**	**ND** **ND** **ND** **ND** **ND** **ND** **ND** **ND** **ND** **ND** **ND** **44.90** **1.28** **1.43** **ND** **ND** **ND** **ND** **ND** **ND** **ND**	**ND** **ND** **ND** **ND** **ND** **ND** **ND** **ND** **ND** **7.14** **ND** **41.03** **3.28** **7.36** **ND** **ND** **4.32** **ND** **ND** **ND** **ND**	**ND** **0.15** **0.70** **0.75** **ND** **6.20** **0.26** **0.45** **ND** **2.06** **0.80** **32.02** **ND** **3.61** **0.32** **0.12** **0.21** **ND** **0.11** **ND** **ND**	**4.09** **ND** **ND** **ND** **ND** **ND** **ND** **ND** **ND** **10.37** **ND** **33.67** **ND** **13.25** **ND** **ND** **ND** **ND** **ND** **ND** **ND**	**ND** **ND** **ND** **ND** **ND** **ND** **ND** **ND** **ND** **0.96** **1.37** **53.59** **0.64** **5.96** **ND** **1.22** **0.71** **ND** **ND** **ND** **ND**
Σ SFA		**44.56**	**54.60**	**51.20**	**58.17**	**50.34**	**54.35**	**47.61**	**63.13**	**48.76**	**61.38**	**64.44**
Nonanoic acid	**C9:1**	**ND**	**28.02**	**ND**	**ND**	**ND**	**0.09**	**ND**	**ND**	**0.28**	**ND**	**ND**
Palmitoleic acid	**C16:1 n-7**	**5.19**	**ND**	**0.41**	**3.86**	**3.59**	**5.33**	**8.46**	**7.92**	**3.57**	**9.25**	**8.39**
Oleic acid	**C18:1 n-9**	**22.82**	**ND**	**38.42**	**34.79**	**32.69**	**37.43**	**34.27**	**9.69**	**42.17**	**16.66**	**15.88**
Gondoic acid	**C20:1**	**ND**	**ND**	**0.56**	**ND**	**ND**	**0.16**	**1.31**	**2.34**	**0.54**	**ND**	**1.39**
Σ MUFA		**28.01**	**28.02**	**39.39**	**38.03**	**36.28**	**43.01**	**44.04**	**19.95**	**46.56**	**21.08**	**25.66**
Linoleic acid Stearidonic acid Eicosatrienoic acid Arachidonic acid	**C18:2 n-6** **C18:4 n-3** **C20:3 n-3** **C20:4 n-6**	**ND** **ND** **ND** **ND**	**6.78** **10.15** **0.32** **ND**	**3.63** **3.22** **ND** **2.48**	**ND** **ND** **ND** **ND**	**ND** **ND** **ND** **ND**	**2.16** **0.10** **ND** **0.38**	**2.15** **0.34** **ND** **0.69**	**ND** **ND** **ND** **ND**	**3.19** **0.26** **ND** **0.09**	**1.81** **ND** **ND** **ND**	**ND** **1.64** **ND** **0.98**
Σ PUFA		**ND**	**17.25**	**9.33**	**ND**	**ND**	**2.64**	**3.18**	**ND**	**3.54**	**1.81**	**2.64**
Σ TFAs		**72.57**	**99.87**	**99.92**	**96.20**	**86.6**	**100.0**	**94.83**	**83.08**	**98.86**	**84.64**	**92.74**

Values are given as percent (%) of total fatty acids. Each value is the mean of three replicates ± standard deviation. *SFA*, saturated fatty acid; *PUFA*, polyunsaturated fatty acid; *MUFA*, monounsaturated fatty acid; *TFAs*, total fatty acids; *ND*, not detected

### Predicting biodiesel quality parameters by fatty acid composition

The biodiesel quality and properties are changed by fatty acid composition. Ten crucial biodiesel parameters were estimated for *C. vulgaris* treated with Co(NO_3_)_2_∙6H_2_O, MnCl_2_, and ZnSO_4_∙7H_2_O in addition to an untreated control culture. The predicted attributes together with the associated international biodiesel specifications are listed in Table 2.

**Table 2:** Predicted biodiesel properties from FAME profiles of *Chlorella vulgaris*

Heavy metals	Concentration	CN	SV (mg KOH g^−1^)	IV (g I_2_ 100 g^−1^ fat)	DU (wt., %)	LCSF (wt., %)	CFPP (°C)	CP (°C)	PP (°C)	OS (h)	FP (min)
	**Control**	**78.68**	**143.77**	**24.49**	**44.20**	**8.79**	**11.15**	**12.74**	**7.02**	**6.07**	**189.36**
Zn (mM)	**0.2**	**58.87**	**203.09**	**63.38**	**62.52**	**7.24**	**6.26**	**12.85**	**7.13**	**5.37**	**185.76**
	**0.4**	**63.36**	**184.15**	**55.68**	**58.05**	**8.02**	**9.10**	**13.47**	**7.80**	**5.54**	**174.85**
	**0.6**	**66.50**	**194.52**	**35.00**	**38.65**	**5.82**	**1.80**	**27.70**	**21**	**6.28**	**150.11**
	**0.8**	**63.15**	**172.80**	**65.27**	**36.28**	**8.00**	**8.45**	**1.88**	**11.50**	**6.38**	**177.33**
Co (µm)	**1**	**63.73**	**202.71**	**42.29**	**48.23**	**8.43**	**9.99**	**15.60**	**10.11**	**5.92**	**172.93**
	**2**	**64.97**	**193.71**	**42.02**	**50.41**	**5.21**	**− 0.124**	**18.63**	**13.40**	**5.21**	**186.13**
	**3**	**74.91**	**167.21**	**17.65**	**19.95**	**14.26**	**28.33**	**16.59**	**11.19**	**7.00**	**189.47**
Mn (mM)	**2**	**62.29**	**205.60**	**46.67**	**53.74**	**5.95**	**2.22**	**12.27**	**6.50**	**5.70**	**176.28**
	**4**	**69.98**	**184.17**	**26.21**	**24.71**	**9.99**	**14.69**	**12.71**	**6.99**	**6.82**	**175.03**
	**6**	**69.74**	**186.79**	**25.47**	**30.94**	**10.63**	**16.90**	**23.20**	**18.36**	**6.58**	**190.12**
Biodiesel standard EN 14214		**≥ 51**	**-**	**≤ 120**	**≤ 137**	**-**	**≤ 5/−20**	**-**	**-**	**≥ 6**	**120**
Biodiesel standard ASTM D97550		**≥ 47**	**-**	**NA**	**-**	**-**	**NA**	**-**	**-**	**≥ 6**	**93**
Biodiesel standard IC15607		**≥ 51**	**-**	**NA**	**-**	**-**	**6/18**	**-**	**3/15**	**≥ 6**	**-**
Previous studies on *Chlorella vulgaris* (Talebi et el. 2013;Francisco et al. 2010; Yang et al. 2016)		**65.77** **44** **78.47**	**217.80** **194** **123.20**	**65** **135.30** **53.91**	**74.15** **116.64** **89.61**	**6.76** **6.71** **6.65**	**4.51** **2.62** **4.14**	**-** **-** **-**	**-** **-** **-**	**-** **-** **-**	**-** **-** **-**

Specifications are in USA, European and Indian standards; *CN*, cetane number; *SV*, saponification value; *IV*, iodine value; *DU*, degree of unsaturation; *LCSF*, long-chain saturated factor; *CFPP*, cold filter plugging point; *CP*, cloud point; *PP*, pour point; *OS*, oxidative stability; *FP*, flash point

The cetane number (CN) is theoretically comparable to the petroleum octane number and is a measure of a fuel’s ability to ignite in a diesel engine. The high amount of saturated and monounsaturated methyl esters may be the cause of the elevated CN value. All of the *C. vulgaris* treated with various heavy metals in the current study displayed high CN values between 58.87 and 74.91 to meet the biodiesel specification standards of Europe EN 14214 and India IC15607 (a minimum CN of 51), and American ASTM D97550 (a minimum CN of 47). The results of the fatty acids profile analysis showed that there were significant amounts of SFA, particularly palmitic acid (C16:0), which ranges from 33.92 to 58.17% of TFA, and stearic acid (C18:0), which varies from 1.37 to 13.25% of TFA and whose carbon chain contains no unsaturated bonds between carbon atoms, preventing it from incorporating any more hydrogen atoms. The results also revealed that the control culture had the greatest CN value (78.68), followed by cultures treated with 3 µM Co(NO_3_)_2_∙6H_2_O (74.91) and 4 mM MnCl_2_ (69.98). It can be assumed that biodiesel derived from algae may have a CN value close to the minimum value in international standards. However, comprehensive data on cetane numbers are not yet available for this biofuel type.

The potassium hydroxide quantity required to neutralize the fatty acids produced by the full hydrolysis of 1 g of oil is indicated by the saponification value (SV). The limits of saponification value (SV) were not stated in biodiesel standards like ASTM D6751, EN 14214, and IS 15607. According to the fatty acid profile results, both treated and untreated *C. vulgaris* exhibited saponification values that ranged from 143.77 mg KOH g^−1^ in the control culture to 205.60 mg KOH g^−1^ at 2 mM MnCl_2_. It is clear from the data that the application of different heavy metals stress on *C. vulgaris* leads to a remarkable increase in the SV value of produced biodiesel.

The fatty acid chain’s number of double bonds and quantity of unsaturation are both determined by the iodine value (IV), which solely depends on the source of the oil. The results indicated that all treated and untreated *C. vulgaris* showed lower iodine values ranging from 17.65 g I_2_/100 g at 3 µm Co(NO_3_)∙6H_2_O to 63.38 g I_2_/100 g at 0.2 mM ZnSO_4_∙7H_2_O. As a result, both treated and untreated *C. vulgaris* biodiesels fall below EN 14214-required maximum IV limits (120 g I2/100 g) for biodiesel. It should be noted that biodiesel with the lowest unsaturated FA content corresponds to the lowest IV value.

The amount of unsaturated FAs is primarily indicated by the degree of unsaturation (DU), which was estimated by the summation of MUFA and PUFA. It is one of the crucial characteristics that affect biodiesel’s oxidative stability. High levels of unsaturation indicated the presence of a high PUFA amount, which had a negative correlation with the biodiesel’s oxidative stability. All of the treated *C. vulgaris* produced DU of FAMEs that ranged from 19.95 wt.% at 3 µM Co(NO_3_)∙6H_2_O to 62.52 wt.% at 0.2 mM ZnSO_4_∙7H_2_O, which is below the EN 142214 maximum limit (≤ 137 wt%).

Based on FA composition, low-temperature characteristics of biodiesel, including cold filter plugging point (CFPP), cloud point (CP), and pour point (PP), were identified. One of the biodiesel’s key characteristics is cold flow performance. Both the European Biodiesel Standard EN 142214 and the Indian Biodiesel Standard IS15607 specified that the CFPP value should be within ≤ 5/− 20 °C and 6/18 °C, respectively. Almost all the treated *C. vulgaris* FAMEs meet these criteria except for 3 µM Co(NO_3_)∙6H_2_O where CFPP was 28.33 °C. As a result of the higher amount of SFA, mostly palmitic acid (C16:0) appeared at a level exceeding 32.04% in treated *C. vulgaris*, the highest temperature of CFPP may be present. Although PUFA may enhance CFPP characteristics, they may also have an impact on the fuel oxidative stability as signified by Fig. 4.

One of the main technical obstacles to biodiesel’s more widespread use and commercialization has been its unfavorable qualities at low temperatures. The lowest temperature at which the soluble biodiesel constituent crystallizes out of the solution is known as cloud point (CP). Low-temperature performance issues could lead to filter blockage from wax buildup and engine starvation from reduced fuel delivery. Because of the significant temperature and seasonal changes, both the American and European biodiesel standards have included a cloud point specification. The findings of this investigation revealed that the CP values for treated *C. vulgaris* ranged from 1.88 °C in 0.8 mM ZnSO_4_∙7H_2_O to 27.70 °C in 0.6 mM ZnSO_4_∙7H_2_O, with the latter showing the highest cloud point because of its greater saturated fatty acids (64.44% of TFA).

The pour point (PP) is an indicator of the diesel gelling point since it is the lowest temperature at which fuel solidifies, loses flow performance, and becomes unpumpable. The findings of this study indicated that the investigated *C. vulgaris* had PP values ranging from 6.50 to 18.36 °C. While the Indian standard stipulates a minimum of 3 °C for winter and 15 °C for summer, neither the US nor European biodiesel standards contained values for PP. Most of the treated *C. vulgaris* FAMEs successfully meet these criteria according to Indian standard (IS 15607) except 0.6 mM ZnSO_4_∙7H_2_O and 6 mM MnCl_2_ which have PP values of 21 and 18.36 °C, respectively.

Long-chain saturation factor (LCSF) influences important biodiesel properties like CN, IV, OS, and CFPP. The higher proportion of the long-chain fatty acids will affect the low-temperature characteristics since long-chain fatty acids typically have higher temperatures of precipitation than shorter-chain fatty acids. The results obtained in this investigation elucidated that as a result of the higher content of palmitic acid (C16:0), *C. vulgaris* culture treated with 3 µM Co(NO_3_)_2_∙6H_2_O displayed the highest LCSF value (14.26 wt.%), whereas 2 µM Co(NO_3_)_2_∙6H_2_O showed the lowest value (5.21 wt.%).

High levels of polyunsaturated FAs, which have the propensity to oxidize quickly, have an effect on biodiesel’s oxidation stability (OS), and it has a negative influence on storage stability which is crucial for fuel applications. Due to the low PUFA content, the oxidative stability of treated *C. vulgaris* FAMEs varied within a constrained range of 5.21 to 7.00 h.

The flash point (FP) is the minimum temperature at which fuel will start to evaporate and combine with air to generate an ignitable combination. The minimum requirement level is 93 °C in the US standard (ASTM D6751-02) compared to at least 120 °C in the European biodiesel specification (EN 142214). Higher values of the flash point make fuels safer for storage, handling, and transportation without immediately affecting combustion. The findings revealed that all treated *C. vulgaris* had estimated FP temperatures that ranged from 150.11 to 190.12 °C, exceeding the established biodiesel limits. According to data, *C. vulgaris* biodiesel has high FP which makes it highly safe for use, storage, and transfer.

**Fig. 7:**
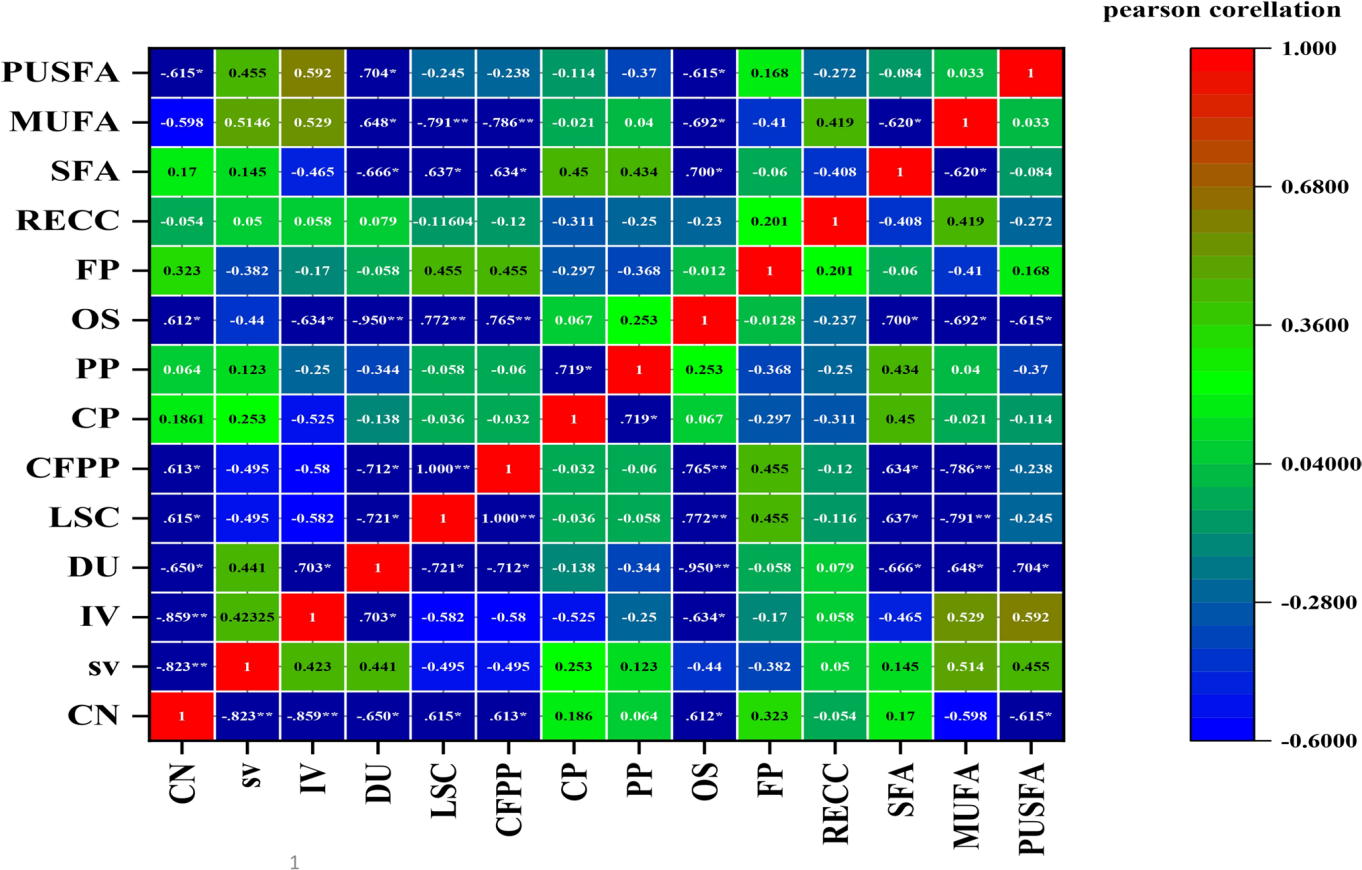
Heat map illustrates Pearson correlation between fatty acid profile of *chlorella vulgaris* under effect of different concentrations of heavy metal and biodiesel properties

Heat map (Fig. 7) illustrates Pearson correlation between the fatty acid profile of *Chlorella vulgaris* under the effect of different concentrations of heavy metal and biodiesel properties. Saturated fatty acid (SFA) of the selected alga shows a positive correlation effect on CN, SV, CP, PP, LSCF, CSPP, and OS (*r* = 0.170, 0.150, 0.450, 0.430, 0.637*, 0.634*, and 0.700*, respectively at *p* < 0.05); in contrast, saturated fatty acid (SFA) was highly negatively correlated with IV, DU, RECC, and FP, respectively at *r* = − 0.470, − 0.666*, − 0.410, and − 0.06 at *p* < 0.05). Monounsaturated fatty acid was positively correlated with SV, DU, LSCF, PP, and RECC (*r* = 0.51, 0.53, 0.648*, 0.04, and 0.42), while CN, LSCF, CP, PP, OS, and FP were negatively correlated with MUFA. SV, IV, DU, and FP were affected positively by the increase of polyunsaturated fatty acid (PUFA) (*r* = 0.46, 0.59, 0.704, and 0.17, respectively, at *p* < 0.05) but it is highly negatively correlated with CN, LSCF, CFPP, CP, PP, OS, and RECC, respectively (*r* = − 0.615* and − 0.25, − 0.24, − 0.11, − 0.37, 0.615*, and − 0.27 at *p* < 0.05).

## Discussion

*Chlorella vulgaris* was evaluated as a potential feedstock for biodiesel production and for manufacturing high-quality biofuel from algae. To assess their impact on growth and metabolic activities, *Chlorella vulgaris* cultures were exposed to various concentrations of the heavy metal, including MnCl_2_ (2, 4, and 6 mM), Co (NO_3_)_2_∙6H_2_O (1, 2, and 3 µM), and ZnSO_4_∙7H_2_O (0.2, 0.4, 0.6, and 0.8 mM). *Chlorella* is particularly appealing since it exhibits great tolerance to a variety of harsh environmental conditions in addition to being similar to vegetable oils; *Chlorella* has more saturated and unsaturated fatty acids (Pulz and Gross 2004). The majority concentrations of the heavy metals have enhanced the growth of *C. vulgaris*. The greatest growth stimulation was recorded at 0.2 mM Zn^2+^, where dry weight was enhanced by 91.82% and the biomass increased by 108.97% in comparison to the control. However, *C. vulgaris* growth showed a non-recoverable drop at the low concentration of 1 µm Co^2+^. These findings concur with those obtained by Priyadarshini et al. (2019), who discovered that *C. vulgaris* is a pioneering species that can survive in harsh environments including those contaminated with heavy metals. The importance of manganese as a necessary component of numerous metalloenzymes, vitamins, and proteins that play crucial roles in metabolism could be used to elucidate growth improvement at higher Mn^2+^ concentrations (Battah et al. 2015). Co^2+^ substitution with Zn^2+^ in certain metalloenzymes may cause growth promotion at low Co^2+^ concentrations (Price and Morel 1990). Whitton (1970) reported that lower Zn^2+^ concentrations would encourage algal growth, while higher concentrations would completely inhibit it. The homeostatic regulation system in microalgae under heavy metal stress produces an overcompensation effect that causes activation of metabolism and antioxidant systems to counteract the toxicity (also known as the hermetic effect) (Spoljaric et al. 2011). Moreover, some heavy metals may have acted as nutrients to aid in the growth of the microalgae, adding another source of nutrients to the medium’s previously present sources. For instance, the functioning of mitochondria and chloroplasts, DNA synthesis, photosynthetic electron transport, and other processes all depend on the cofactors Cu^2+^ and Zn^2+^, which are present in enzymes (Einicker Lamas et al. 2002). Furthermore, Afkar et al. (2010) demonstrated that zinc is not only a crucial mineral for algal metabolism but can also be hazardous when used in higher amounts than necessary. The interruption of cell division, inactivation of proteins, disorganization of mitochondria and chloroplast, rupture of the chloroplast envelop, membrane integrity deterioration, and even lysis by heavy metals could all be reasons why concentrations of heavy metals inhibit growth (Einicker Lamas et al. 2002; Pinto et al. 2003; La Rocca et al. 2009).

The finding of this study regarding photosynthetic pigments showed that treatment with various heavy metals had a substantial impact on the cell contents of these pigments in *C. vulgaris*. Except for 1 µm Co^2+^, the majority of heavy metal concentrations significantly increased the amount of photosynthetic pigments. This finding was in accordance with numerous previous published results (ElSheekh et al. 2003; Osman et al. 2004; Badawy et al. 2005; Romera et al. 2007; Luo et al. 2015; Mohy ElDin 2016). Similarly to this, Rai and Sharma (2006) found that algae grown in the presence of heavy metals had significantly higher chlorophyll a and beta-carotene levels. Saçan and Balcıoğlu (2006) observed that low concentrations of heavy metal treatments dramatically increased the chlorophyll contents of algae. According to Fisher et al. (1981), *Asterionella japonica* has higher total chlorophyll due to decreased Zn^2+^ levels.

The principal organic substances produced within algal cells by photosynthetic activity are carbohydrates. The presence of various heavy metal concentrations was reported to have an impact on the examined alga’s carbohydrate content. In algal cultures treated with various heavy metals, *C. vulgaris* total carbohydrate content rises markedly. Our findings were consistent with Sharma and Agrawal (2005) observations of considerable enhancement in the carbohydrates of algae under heavy metal stress. It appeared to be a suitable strategy that the massive production of soluble carbohydrates may use to detoxify heavy metal stress (Arunakumara and Zhang 2008). Costa and Spitz (1997) demonstrated that the amounts of carbohydrate production improved with low Mn concentrations.

The kind of heavy metal and dose had an impact on how *C. vulgaris* responded to various heavy metals in the form of total protein. The impact of heavy metals on *C. vulgaris* shows that the amount of protein varies depending on the dose of the element under study. Reactive oxygen species can be overproduced due to heavy metals, which can lead to harmful oxidation and protein degradation (Doganlar et al. 2012). There are previous reports of both increases and decreases in the total protein content of algae during heavy metal stress (Vajpayee et al. 2000). Furthermore, protein breakdown by oxidative damage may be the cause of the declining protein content in the heavy metal–treated algae. Nevertheless, the rise in certain stress-related proteins, including enzymes included in antioxidant metabolism and photoheating biosynthesis, was likely the reason for the increases in total protein content brought on by heavy metals (Doganlar et al. 2012). One theory is that the buildup of protein fractions at lower concentrations of heavy metals may enable algae to counteract their harmful effects, or that increasing respiration may encourage the use of carbohydrates for protein accumulation (Osman et al. 2004). The reduction in protein synthesis can be explained by a lack of carbon skeleton due to a poor photosynthetic rate (Afkar et al. 2010).

Green microalgae were shown to associate higher cellular lipid accumulation with various environmental stress situations. High lipid content is essential for biofuel production to be commercially viable (Li et al. 2011). High lipid production is required in appropriate micro-algal species, either by supplying high baseline lipid content or by accumulating significant amounts of lipids (Rodolfi et al. 2009). The present study shows that the increase in Mn^2+^ concentration leads to decreases in lipid yields while increases in Co^2+^ and Zn^2+^ concentrations lead to a significant increase in lipid production. In this regard, Napan et al. (2015) found that there was a favorable zone for heavy metal concentration for optimum lipid production and the difference in this zone depends on the heavy metal type and algal species (Solovchenko et al. 2009; Menon et al. 2013). The disturbance of algal metabolism caused by the inactivation of the photosynthetic apparatus, which causes the synthesis of lipids as storage molecules instead of carbohydrates, may be the cause of the increased lipid content at high concentrations of heavy metals (Chia et al. 2013). Due to the increased creation of reactive oxygen species (ROS), a byproduct of aerobic metabolism, heavy metals can cause cellular oxidative stress (Zhang et al. 2013). It has been hypothesized that under stress conditions, microalgae accumulate more lipids as a defense against ROS, where excess cytoplasmic lipid droplets act as a sink to sequester ROS-scavenger molecules, thus increasing their production (Solovchenko et al. 2009). According to the stress mechanism described above, heavy metals may have directly contributed to stress by catalyzing the production of ROS molecules by redox-active Co (II) and Mn (II) or by consuming the antioxidant pool (ROS-scavenger molecules) by non-redox-active Zn (II) (Einicker Lamas et al. 2002; Pinto et al. 2003). The endoplasmic reticulum disruption and chloroplast damage caused by heavy metals stress may be the cause of the lower lipid production (Einicker Lamas et al. 2002; Pinto et al. 2003). Future research should focus on processes that increase lipid and biomass yields since they have the potential to be profitable for the production of the algal-based biodiesel industry.

### Fatty acid composition

The primary criteria for choosing the algal strains for biodiesel production are higher lipid levels and volumetric lipid productivity (Nascimento et al. 2013). For this reason, a thorough examination of fatty acid composition was carried out in the current study. The fatty acid profile of *C. vulgaris* revealed the appearance of palmitic acid (C16:0), palmitoleic acid (C16:1), stearic acid (C18:0), oleic acid (C18:1), γ-linolenic acid (C18:3), and behenic acid (C22:0). The primary fatty acid species found in vegetable oils were previously identified as palmitic (C16:0), stearic (C18:0), oleic (C18:1), linoleic (C18:2), and linolenic (C18:3) (Hoekman et al. 2012) and in other microalgae species (Dasgupta et al. 2015). In accordance with these results, the greatest levels of palmitic acid, stearic acid, and myristic acid content were found in treated strains of *C. vulgaris* (Francisco et al. 2010; AbouShanab et al. 2011). It was fascinating to note that treated algal cultures have a significant percentage of saturated fatty acids constituted by stearic acid (C18:0) and palmitic acid (C16:0). Researchers found that the NOx emissions increased when biodiesel was utilized instead of fossil diesel (Jagtap et al. 2020). When gasoline has more saturated fatty acids (SFA) than unsaturated fatty acids (UFA), NOx exhaust emissions are decreased. According to Schönborn et al. (2009), SFA generates less NOx than UFA. SFA molecules have superior fuel qualities, such as a higher cetane number, lower density and viscosity values, and a faster combustion rate. Reduced ignition time as a result will lessen the production of thermal NOx (Schönborn et al. 2009). Another benefit of SFA is that they have greater cetane numbers (CN) than UFA, suggesting superior biodiesel ignition quality and improved oxidative stability (Knothe 2009).

The current study’s findings support the earlier revelation that higher chain fatty acids, such as C18:2 and C18:3, were found to be present in lower concentrations in algal oils than in vegetable oils (Knothe 2011). The treated strains of *C. vulgaris* had the highest concentrations of palmitic acid, stearic acid, and myristic acid (Francisco et al. 2010). The findings of the current investigation demonstrated that FA composition showed the highest proportion of SFA than MUFA and PUFA. Previous studies showed that the best biodiesel should have less SFA and PUFA than MUFA to reduce oxidative stability and cold flow issues (Knothe 2009; Hoekman et al. 2012). According to Ramos et al. (2009), cells that are resistant to growth inhibitors develop an excess of PUFA production as a result of the inhibitor’s influence on fatty acid desaturation. One indicator of lipid peroxidation is the particular suppression of linolenic acid under environmental stress (ElShintinawy and ElShourbagy 1997). According to Hoekman et al. (2012), Mn, Co, and Zn had an impact on raising the ratio of unsaturated to saturated fatty acids, which increased the fluidity of membranes. Membranes can become more or less fluid according to the length of the fatty acid chain (Knothe 2002). The results of this study revealed that most treated algal culture contains a high amount of γ-linolenic acid which led to high biodiesel properties according to European standards (EN 14214) that specify a limit of 12% for high-quality biodiesel.

### Fuel properties

The composition of the fatty acids greatly affects the quality of fuel. Long-chain fatty acids (C16–C18) are desired because the cetane number (CN), the heat of combustion, and viscosity all rise with increasing chain length (Francisco et al. 2010). Fuels containing significant quantities of saturated fatty acids have higher CN values (Gopinath et al. 2009). Better ignition quality is correlated with greater CN. Additionally, saturated esters with high CN values have poor cold flow characteristics. Unsaturated, particularly polyunsaturated, fatty acids have lower melting points than saturated fatty acids, which is preferable for enhanced low-temperature characteristics but unfavorable for fuel because of their low CN and decreased oxidative stability (Knothe 2009). Thus, for good biodiesel quality, saturated and unsaturated FAMEs should have an optimal balance (Islam et al. 2013). In this study, the characteristics of *C. vulgaris* biodiesel were compared with Indian standards (IS 1560), European standards (EN 14214), and American standards (ASTM D-6751). CN values range between 58.87 and 78.68, meeting the minimum value of 51 specified by the international standard (Hoekman et al. 2012). More or less similar results were obtained previously on *C. vulgaris* which reported that CN ranges from 44 to 78.47 (Francisco et al. 2010; Talebi et al. 2013). This is caused by the high concentration of saturated fatty acids (SFA), particularly stearic acid, whose carbon chain lacks unsaturated links between carbon atoms and is therefore unable to incorporate any additional hydrogen atoms (Anahas and Muralitharan 2015).

The saponification value (SV) in this study ranges from 143.77 to 202.71 mg KOH g^−1^*.* These results agreed with the previous study on *C. vulgaris* which reported that SV ranges from 123.20 to 217.80 KOH g^−1^ (Francisco et al. 2010; Talebi et al. 2013). SV is negatively correlated with fatty acid molecular weight. The lower the molecule mass, the greater will be the SV value (Dasgupta et al. 2015). The maximum SV value was not specified in American, European, and Indian biodiesel standards (Predojević et al. 2012).

The quantity of unsaturation in FAs is determined by the iodine value (IV), which also increases the number of double bonds in the fatty acid chain and only depends on the origin of the oil (Ramos et al. 2009). High IV may lead to increased sensitivity to oxidative attack, the production of deposits, and loss of the lubricity of the biodiesel (Francisco et al. 2010). The findings showed that all of the *C. vulgaris*-treated cultures had iodine values that ranged from 17.65 to 63.38 g I_2_/100 g, which is within the maximum limit of 120 g I_2_/100 g for biodiesel requirement standards. It should be noted that the biodiesel with the lowest unsaturated fatty ester concentration has the lowest IV. More or less similar lower values of IV were recorded for *C. vulgaris* ranging from 53.91 to 65.52 I_2_/100 g in previous studies (Miriam et al. 2017).

One of the key factors affecting the oxidative stability of biodiesel is the degree of unsaturation (DU) (Francisco et al. 2010). Large amounts of PUFA were present due to high unsaturation levels, which related negatively with the biodiesel oxidative stability (Mandotra et al. 2014) but positively with its mechanical fuel qualities due to a decrease in viscosity values (Francisco et al. 2010). DU of FAMEs obtained from *C. vulgaris* varied within a range of 19.95 to 62.52 (wt. %), which is less than the upper limit permitted by European standards (137 wt%) (Ramos et al. 2009). These values were below the values obtained in previous studies which indicate that DU ranges from 74.10 to 116.60 (wt. %) in *C. vulgaris* (Francisco et al. 2010; Talebi et al. 2013).

The low-temperature flow characteristics of biodiesel are one of the main issues with its utilization. In this study, the FA composition was used to estimate the low-temperature flow characteristics of biodiesel, including cold filter plugging point (CFPP), cloud point (CP), and pour point (PP). According to the seasonal and temperature fluctuations, Knothe (2009) proposed that every nation should specify the standard biodiesel limitations for the cold flow characteristics. The pour point (PP), the lowest temperature at which biodiesel will still flow, is used to measure the flow characteristics (Knothe 2013). Biodiesel made from fats or oils that contain a large quantity of saturated fatty constituents has greater PPs (Imahara et al. 2006). The generated biodiesel in this investigation had PPs that ranged from 7.02 to 21 °C, which is within the international standard-required range. As a result, it is best suited for areas with a moderate climate. The *C. vulgaris* biodiesel flash point (FP) ranges from 150.11 to 190.12 °C, which is higher than those of American standards (120 °C) and Petro diesel. As a result of having lower flammability, biodiesel with higher flash points is safer to handle, store, and transport (Barabás and Todoruţ 2011).

The lowest temperature at which the least soluble biodiesel component crystallizes out of the solution is known as the cloud point (CP). Cold flow performance is one of biodiesel’s key characteristics. Low-temperature performance issues might lead to filter blockage from wax production and engine starvation from reduced fuel flow. Generally, the occurrence of a higher quantity of long-chain and saturated FA in biodiesel leads to poor cold flow characteristics (Hoekman et al. 2012). Because of the significant seasonal and temperature changes, none of the biodiesel standards (ASTM D6751 and EN 1424) have specified cloud point (CP) limits. The findings of this investigation revealed that the estimated cloud point for treated *C. vulgaris* biodiesels ranged from 1.88 °C in 0.8 mM ZnSO_4_∙7H_2_O to 27.70 °C in 0.6 mM ZnSO_4_∙7H_2_O. Due to their increased amount of saturated fatty acids (64.44% of TFA), *C. vulgaris* cultures treated with 0.6 mM zinc sulfate had the highest cloud point. These results are in consistent with previous CP value results of − 2.47 to 9.71 °C (Anahas and Muralitharan 2015).

One of the most important characteristics of biodiesel is the cold filter plugging point (CFPP). The CFPP value was specified to be within ≤ 5/− 20 °C by the European Biodiesel Standard EN 142214 and 6/18 °C by the Indian Biodiesel Standard IS15607. Most of the treated *C. vulgaris* FAMEs meet these criteria except for 3 µM Co (NO_3_)∙6H_2_O where CFPP was 28.33 °C. Due to the large proportion of saturated fatty esters in biodiesel, the CFPP value rises proportionately, and this increase is especially noticeable for long-chain fatty esters. Thus, the CFPP value was required to fall between − 5 and – 13 °C according to American biodiesel standards (Ramos et al. 2009). Nevertheless, the occurrence of PUFA may enhance the cold flow characteristics but have an impact on the oxidative stability of fuel as shown by IV (Giakoumis 2013).

Long-chain saturation factor (LCSF) influences important biodiesel properties including cetane number (CN), iodine value (IV), oxidation stability (OS), and cold filter plugging point (CFPP). The long-chain fatty acids typically have a higher temperature of precipitation than shorter-chain fatty acids; hence, the larger proportion of these fatty acids will impact the low-temperature characteristics (Anahas and Muralitharan 2018). The highest LCSF value (14.26 wt.%) in this investigation came from *C. vulgaris* culture treated with 3 µM Co(NO_3_)_2_∙6H_2_O, whereas the lowest value (5.21 wt.%) came from a culture treated with 2 µM Co(NO_3_)_2_∙6H_2_O. Due to the high concentration of palmitic acid (C16:0) in the treated algal cultures, the LCSF value was at its greatest. According to Francisco et al. (2010), long-chain fatty acids affect low-temperature characteristics contrarily.

To meet the ASTM D6751 (3 h) and EN 14214 (6 h) specified values, the oxidative stability (OS) of FAMEs in treated *C. vulgaris* throughout this investigation varied within a small range of 5.21 to 7.00 h. This might be attributed to the low level of PUFA (Ramos et al. 2009). High levels of polyunsaturated FAs can affect the biodiesel oxidation stability, making it unstable to oxidation and having a detrimental effect on storage stability, which is important for fuel applications (Knothe and Dunn 2003; Arias Peñaranda et al. 2013). Earlier research found a negative association between the degree of unsaturation and oxidative stability, notably for polyunsaturated FAs (C18:2, C18:3) (Ramos et al. 2009). This is because the reactive sites on these unsaturated fatty acid chains are particularly vulnerable to assault by free radicals (Knothe and Dunn 2003; Arias Peñaranda et al. 2013).

## Conclusion

The present study was conducted to evaluate the potential use of *Chlorella vulgaris* for biodiesel production. The stress effect of different heavy metals (Mn^2+^, Co^2+^, and Zn^2+^) on the algal cultures enhanced the production of the algal biodiesel. Empirical formulas were used for the assessment of biodiesel fuel properties based on FAME composition. The estimated properties met the prescribed international biodiesel standard criteria.

## Data Availability

The data that support the findings of this study are available on request from the corresponding author.

## References

[CR1] Abomohra A, Wagner M, ElSheekh M, Hanelt D (2013). Lipid and total fatty acid productivity in photoautotrophic fresh water microalgae: screening studies towards biodiesel production. J Appl Phycol.

[CR2] AbouShanab RAI, Hwang JH, Cho Y, Min B, Jeon BH (2011). Characterization of microalgal species isolated from freshwater bodies as a potential source for biodiesel production. Appl Energy.

[CR3] Afkar E, Ababna H, Fathi AA (2010). Toxicological response of the green alga Chlorella vulgaris, to some heavy metals. Am J Environ Sci.

[CR4] Agarwal M, Singh K, Chaurasia SP (2010). Prediction of biodiesel properties from fatty acid composition using linear regression and ANN techniques. Indian Chem Eng.

[CR5] Anahas AMP, Muralitharan G (2015). Isolation and screening of heterocystous cyanobacterial strains for biodiesel production by evaluating the fuel properties from fatty acid methyl ester (FAME) profiles. Bioresour Technol.

[CR6] Anahas AMP, Muralitharan G (2018). Characterization of heterocystous cyanobacterial strains for biodiesel production based on fatty acid content analysis and hydrocarbon production. Energy Convers Manag.

[CR7] Ansari FA, Nasr M, Guldhe A, Rawat SK, Bux F (2020). Techno-economic feasibility of algal aquaculture via fish and biodiesel production pathways: a commercial-scale application. Sci Total Environ.

[CR8] Arias Peñaranda MT, Cristiani Urbina E, Montes Horcasitas C, Esparza García F, Torzillo G, Cañizares Villanueva RO (2013) Scenedesmus incrassatulus CLHE-Si01: a potential source of renewable lipid for high quality biodiesel production. Bioresour Technol 140:158–164. 10.1016/j.biortech.2013.04.080 10.1016/j.biortech.2013.04.080 23688667

[CR9] Arunakumara K, Zhang X (2008). Heavy metal bioaccumulation and toxicity with special reference to microalgae. J Ocean Univ china.

[CR10] Badawy WA, Ismail KM, Fathi AM (2005). Effect of Ni content on the corrosion behavior of Cu–Ni alloys in neutral chloride solutions. Electrochim Acta.

[CR11] Barabás I, Todoruţ IA (2011) Biodiesel quality, standards and properties. In: Montero G, Stoytcheva M (eds) Biodiesel-quality, Emissions and By-products, pp 3–28

[CR12] Battah M, ElAyoty Y, Abomohra A, Abd El-Ghany S, Esmael A (2015). Effect of Mn ^2+^, Co^2+^ and H_2_O_2_ on biomass and lipids of the green microalga *Chlorella vulgaris* as a potential candidate for biodiesel production. Ann Microbiol.

[CR13] Bórawski P, Bełdycka Bórawska A, Szymańska EJ, Jankowski KJ, Dubis B, Dunn JW (2019). Development of renewable energy sources market and biofuels in The European Union. J Clean Prod.

[CR14] Chia MA, Lombardi AT, Melão MDGG, Parrish CC (2013). Effects of cadmium and nitrogen on lipid composition of Chlorella vulgaris (Trebouxiophyceae, Chlorophyta). Eur J Phycol.

[CR15] Chisti Y (2007). Biodiesel from microalgae. Biotechnol Adv.

[CR16] Costa G, Spitz E (1997). Influence of cadmium on soluble carbohydrates, free amino acids, protein content of in vitro cultured Lupinus albus. Plant Sci.

[CR17] Dasgupta CN, Suseela MR, Mandotra SK, Kumar P, Pandey MK, Toppo K, Lone JA (2015). Dual uses of microalgal biomass: an integrative approach for biohydrogen and biodiesel production. Appl Energy.

[CR18] Dębowski M, Zieliński M, Grala A, Dudek M (2013). Algae biomass as an alternative substrate in biogas production technologies. Renew Sustain Energy Rev.

[CR19] Demirbas A, Demirbas MF (2011). Importance of algae oil as a source of biodiesel. Energy Convers Manag.

[CR20] Doganlar ZB, Cakmak S, Yanik T (2012). Metal uptake and physiological changes in Lemna gibba exposed to manganese and nickel. Int J Biol.

[CR21] Dučić T, Polle A (2007). Manganese toxicity in two varieties of Douglas fir (Pseudotsuga menziesii var. viridis and glauca) seedlings as affected by phosphorus supply. Funct Plant Biol.

[CR22] Einicker Lamas M, Mezian GA, Fernandes TB, Silva FLS, Guerra F, Miranda K, Oliveira MM (2002). Euglena gracilis as a model for the study of Cu2+ and Zn2+ toxicity and accumulation in eukaryotic cells. Environ Pollut.

[CR23] ElAgawany NI, Kaamoush MIA (2022). Algal sensitivity to nickel toxicity in response to phosphorus starvation. Sci Rep.

[CR24] ElSheekh MM, El Naggar AH, Osman MEH, El-Mazaly E (2003). Effect of cobalt on growth, pigments and the photosynthetic electron transport in Monoraphidium minutum and Nitzchia perminuta. Brazilian J Plant Physiol.

[CR25] ElShintinawy F, ElShourbagy MN (1997). Recovery of photosystem 2 and membrane lipid composition in triazine-treated soybean seedlings by vitamins. Biol Plant.

[CR26] Fatma T, Sultan S (1999) Significance of n‐3 polyunsaturated fatty acids and algal potential as its source. Cyanobacterial algal Metab Environ Biotechnol Narosa Publ house, New Delhi, India 49-60

[CR27] Fisher NS, Jones GJ, Nelson DM (1981). Effects of copper and zinc on growth, morphology, and metabolism of Asterionella japonica (Cleve) 1. J Exp Mar Bio Ecol.

[CR28] Folch J, Lees M, Stanley GHS (1957). A simple method for the isolation and purification of total lipides from animal tissues. J Biol Chem.

[CR29] Francisco EC, Neves DB, Jacob-Lopes E, Franco TT (2010). Microalgae as feedstock for biodiesel production: carbon dioxide sequestration, lipid production and biofuel quality. J Chem Technol Biotechnol.

[CR30] Furniss BS, Hannaford AJ, Smith PWG, Tatchell AR (1989) Vogel’s text book of practical organic chemistry, 5th edn. Longman, New York

[CR31] Giakoumis EG (2013). A statistical investigation of biodiesel physical and chemical properties, and their correlation with the degree of unsaturation. Renew Energy.

[CR32] Gopinath A, Puhan S, Nagarajan G (2009). Relating the cetane number of biodiesel fuels to their fatty acid composition: a critical study. Proc Inst Mech Eng Part D J Automob Eng.

[CR33] Hoekman SK, Broch A, Robbins C, Ceniceros E, Natarajan M (2012). Review of biodiesel composition, properties, and specifications. Renew Sustain energy Rev.

[CR34] Imahara H, Minami E, Saka S (2006). Thermodynamic study on cloud point of biodiesel with its fatty acid composition. Fuel.

[CR35] Islam MA, Magnusson M, Brown RJ, Ayoko GA, Nabi MN, Heimann K (2013). Microalgal species selection for biodiesel production based on fuel properties derived from fatty acid profiles. Energies.

[CR36] Jagtap SP, Pawar AN, Lahane S (2020). Improving the usability of biodiesel blend in low heat rejection diesel engine through combustion, performance and emission analysis. Renew Energy.

[CR37] John RP, Anisha GS, Nampoothiri KM, Pandey A (2011). Micro and macroalgal biomass: a renewable source for bioethanol. Bioresource Technol.

[CR38] Knothe G (2002). Structure indices in FA chemistry. How relevant is the iodine value?. J Am Oil Chem Soc.

[CR39] Knothe G (2007). Some aspects of biodiesel oxidative stability. Fuel Process Technol.

[CR40] Knothe G (2009). Improving biodiesel fuel properties by modifying fatty ester composition. Energy Environ Sci.

[CR41] Knothe G (2011). A technical evaluation of biodiesel from vegetable oils vs. algae. Will algae-derived biodiesel perform?. Green Chem.

[CR42] Knothe G, Dunn RO (2003). Dependence of oil stability index of fatty compounds on their structure and concentration and presence of metals. J Am Oil Chem Soc.

[CR43] Knothe G (2013) Production and properties of biodiesel from algal oils. In: Borowitzka, M., Moheimani, N. (eds) Algae for Biofuels and Energy. Developments in Applied Phycology, vol 5. Springer, Dordrecht. 10.1007/978-94-007-5479-9_12

[CR44] La Rocca N, Andreoli C, Giacometti GM, Rascio N, Moro I (2009). Responses of the Antarctic microalga Koliella antarctica (Trebouxiophyceae, Chlorophyta) to cadmium contamination. Photosynthetica.

[CR45] Leganés F, Sánchez-Maeso E, Fernández Valiente E (1987). Effect of indoleacetic acid on growth and dinitrogen fixation in cyanobacteria. Plant Cell Physiol.

[CR46] Li Y, Han D, Sommerfeld M, Hu Q (2011). Photosynthetic carbon partitioning and lipid production in the oleaginous microalga *Pseudochlorococcum* sp. (Chlorophyceae) under nitrogen-limited conditions. Bioresource Technol.

[CR47] Lowery OH, Rosebrough NJ, Farr AL, Randall RJ (1951). Protein measurement with the Folin phenol reagent. J Biol Chem.

[CR48] Luo X, Su P, Zhang W (2015). Advances in microalgae-derived phytosterols for functional food and pharmaceutical applications. Mar Drugs.

[CR49] Mandotra SK, Kumar P, Suseela MR, Ramteke PW (2014). Fresh water green microalga Scenedesmus abundans: a potential feedstock for high quality biodiesel production. Bioresource Technol.

[CR50] Marker AFH (1972). The use of acetone and methanol in the estimation of chlorophyll in the presence of phaeophytin. Freshw Biol.

[CR51] Menon KR, Balan R, Suraishkumar GK (2013). Stress induced lipid production in Chlorella vulgaris: relationship with specific intracellular reactive species levels. Biotechnol Bioeng.

[CR52] Metzner H, Rau H, Senger H (1965). Untersuchungen zur synchronisierbarkeit einzelner pigmentmangel-mutanten von Chlorella. Planta.

[CR53] Miriam LRM, Raj RE, Kings AJ, Visvanathan MA (2017). Identification and characterization of a novel biodiesel producing halophilic Aphanothece halophytica and its growth and lipid optimization in various media. Energy Convers Manag.

[CR54] Mohy ElDin S (2016). Effects of heavy metals (copper, cobalt and lead) on the growth and photosynthetic pigments of the green alga Chlorella pyrenoidosa H. chick. Catrina Int J Environ Sci.

[CR55] Mukhopadhyay MJ, Sharma A (1991). Manganese in cell metabolism of higher plants. Bot Rev.

[CR56] Munda IM, Hudnik V (1988). The effects of Zn, Mn, and Co accumulation on growth and chemical composition of Fucus vesiculosus L under different temperature and salinity conditions. Mar Ecol.

[CR57] Myśliwa Kurdziel B, Prasad MN V, Strzałtka K (2004) Photosynthesis in heavy metal stressed plants. In: Heavy Metal Stress in Plants. Springer, pp 146–181. 10.1007/978-3-662-07743-6_6

[CR58] Napan K, Teng L, Quinn JC, Wood BD (2015). Impact of heavy metals from flue gas integration with microalgae production. Algal Res.

[CR59] Nascimento IA, Marques SSI, Cabanelas ITD, Pereira SA, Druzian JI, de Souza CO, Nascimento MA (2013). Screening microalgae strains for biodiesel production: lipid productivity and estimation of fuel quality based on fatty acids profiles as selective criteria. Bioenergy Res.

[CR60] Nichols HW (1973) Growth media – freshwater. In Stein, J. (Ed.) Handbook of phycological methods, culture methods and growth measurements, Camb Univ Press pp 7-24

[CR61] Osman MEH, ElNaggar AH, ElSheekh MM, ElMazally EE (2004). Differential effects of Co^2+^ and Ni2+ on protein metabolism in *Scenedesmus obliquus* and *Nitzschia perminuta*. Environ Toxicol Pharmacol.

[CR62] Osman MEH, AboShady AM, Elshobary ME, Abd ElGhafar MO, Abomohra A (2020). Screening of seaweeds for sustainable biofuel recovery through sequential biodiesel and bioethanol production. Environ Sci Pollut Res.

[CR63] Pandey A, Naik MM, Dubey SK (2010). Organic metabolites produced by *Vibrio parahaemolyticus* strain An3 isolated from Goan mullet inhibit bacterial fish pathogens. African J Biotechnol.

[CR64] Pinto E, Sigaud KTCS, Leitao MAS, Okamoto OK, Morse D, Colepicolo P (2003). Heavy metal–induced oxidative stress in algae. J Phycol.

[CR65] Predojević Z, Škrbić B, Đurišić Mladenović N (2012). Transesterification of linoleic and oleic sunflower oils to biodiesel using CaO as a solid base catalyst. J Serbian Chem Soc.

[CR66] Price NM, Morel FMM (1990). Cadmium and cobalt substitution for zinc in a marine diatom. Nature.

[CR67] Priyadarshini E, Priyadarshini SS, Pradhan N (2019). Heavy metal resistance in algae and its application for metal nanoparticle synthesis. Appl Microbiol Biotechnol.

[CR68] Pulz O, Gross W (2004). Valuable products from biotechnology of microalgae. Appl Microbiol Biotechnol.

[CR69] Rai AK, Sharma NK (2006). Phosphate metabolism in the cyanobacterium Anabaena doliolum under salt stress. Curr Microbiol.

[CR70] Ramos MJ, Fernández CM, Casas A, RodríguezL Pérez Á (2009). Influence of fatty acid composition of raw materials on biodiesel properties. Bioresour Technol.

[CR71] Rodolfi L, Chini Zittelli G, Bassi N, Padovani G, Biondi N, Bonini G, Tredici MR (2009). Microalgae for oil: strain selection, induction of lipid synthesis and outdoor mass cultivation in a low-cost photobioreactor. Biotechnol Bioeng.

[CR72] Romera E, González F, Ballester A, Blázquez ML, Munoz JA (2007). Comparative study of biosorption of heavy metals using different types of algae. Bioresour Technol.

[CR73] Saçan MT, Balcıoğlu IA (2006). A case study on algal response to raw and treated effluents from an aluminum plating plant and a pharmaceutical plant. Ecotoxicol Environ Saf.

[CR74] Sarin A, Arora R, Singh NP, Sarin R, Malhotra RK, Kundu K (2009). Effect of blends of palm-Jatropha-Pongamia biodiesels on cloud point and pour point. Energy.

[CR75] Schönborn A, Ladommatos N, Allan R, Williams J, Rogerson J (2009). Effect of the molecular structure of individual fatty acid alcohol esters (biodiesel) on the formation of Nox and particulate matter in the diesel combustion process. SAE Int J Fuels Lubr.

[CR76] Sharma RK, Agrawal M (2005). Biological effects of heavy metals: an overview. J Environ Biol.

[CR77] Shrotri CK, Rathore VS, Mohanty P (1981). Studies on photosynthetic electron transport, photophosphorylation and CO2 fixation in zinc deficient leaf cells of Zea mavs. L J Plant Nutr.

[CR78] Siripornadulsil S, Traina S, Verma DPS, Sayre RT (2002). Molecular mechanisms of proline-mediated tolerance to toxic heavy metals in transgenic microalgae. Plant Cell.

[CR79] Solovchenko AE, Khozin Goldberg I, Cohen Z, Merzlyak MN (2009). Carotenoid-to-chlorophyll ratio as a proxy for assay of total fatty acids and arachidonic acid content in the green microalga Parietochloris incisa. J Appl Phycol.

[CR80] Spoljaric D, Cipak A, Horvatic J, Andrisic L, Waeg G, Zarkovic N, Jaganjac M (2011). Endogenous 4-hydroxy-2-nonenal in microalga Chlorella kessleri acts as a bioactive indicator of pollution with common herbicides and growth regulating factor of hormesis. Aquat Toxicol.

[CR81] Talebi AF, Mohtashami SK, Tabatabaei M, Tohidfar M, Bagheri A, Zeinalabedini M, Bakhtiari S (2013). Fatty acids profiling: a selective criterion for screening microalgae strains for biodiesel production. Algal Res.

[CR82] Tripathi BN, Gaur JP (2006). Physiological behavior of Scenedesmus sp. during exposure to elevated levels of Cu and Zn and after withdrawal of metal stress. Protoplasma.

[CR83] Vajpayee P, Tripathi RD, Rai UN, Ali MB, Singh SN (2000). Chromium (VI) accumulation reduces chlorophyll biosynthesis, nitrate reductase activity and protein content in Nymphaea alba L. Chemosphere.

[CR84] Vogel AI (1975) “A text book of practical organic chemistry”, p. 292, Third ed., English Language Book Society, London

[CR85] Whitton BA (1970). Toxicity of heavy metals to freshwater algae: a review. Phykos.

[CR86] Yemm EW, Willis AJ (1954). The estimation of carbohydrates in plant extracts by anthrone. Biochem J.

[CR87] Zhang Y-M, Chen H, He C-L, Wang Q (2013). Nitrogen starvation induced oxidative stress in an oil-producing green alga Chlorella sorokiniana C3. PLoS One.

